# Identification of cerebral spinal fluid protein biomarkers in Niemann-Pick disease, type C1

**DOI:** 10.1186/s40364-023-00448-x

**Published:** 2023-01-31

**Authors:** Kiersten Campbell, Niamh X. Cawley, Rachel Luke, Katelin E. J. Scott, Nicholas Johnson, Nicole Y. Farhat, Derek Alexander, Christopher A. Wassif, Wenping Li, Stephanie M. Cologna, Elizabeth Berry-Kravis, An Dang Do, Ryan K. Dale, Forbes D. Porter

**Affiliations:** 1 Bioinformatics and Scientific Programming Core, Eunice Kennedy Shriver National Institute of Child Health and Human Development, National Institutes of Health, 10CRC, Rm. 1-3330, 10 Center Dr., Bethesda, MD 20879 USA; 2 Section On Molecular Dysmorphology, Division of Translational Research, Eunice Kennedy Shriver National Institute of Child Health and Human Development, National Institutes of Health, Bethesda, MD USA; 3 Department of Chemistry and Laboratory of Integrative Neuroscience, University of Illinois Chicago, Chicago, IL USA; 4 Rush University Medical Center, Chicago, IL USA; 5 Unit On Cellular Stress in Development and Diseases, Division of Translational Research, Eunice Kennedy Shriver National Institute of Child Health and Human Development, National Institutes of Health, Bethesda, MD USA

**Keywords:** Niemann-Pick disease, Type C1, Proximal extension assay, Biomarkers, Cerebrospinal fluid, CCL18, CALB2, CHI3L1, MIF, ENO2

## Abstract

**Background:**

Niemann-Pick disease, type C1 (NPC1) is an ultrarare, recessive, lethal, lysosomal disease characterized by progressive cerebellar ataxia and cognitive impairment. Although the NPC1 phenotype is heterogeneous with variable age of onset, classical NPC1 is a pediatric disorder. Currently there are no therapies approved by the FDA and therapeutics trials for NPC1 are complicated by disease rarity, heterogeneity, and the relatively slow rate of neurological decline. Thus, identification of disease relevant biomarkers is necessary to provide tools that can support drug development efforts for this devastating neurological disease.

**Methods:**

Proximal extension assays (O-link® Explore 1536) were used to compare cerebrospinal fluid (CSF) samples from individuals with NPC1 enrolled in a natural history study and non-NPC1 comparison samples. Relative expression levels of 1467 proteins were determined, and candidate protein biomarkers were identified by evaluating fold-change and adjusted Kruskal–Wallis test p-values. Selected proteins were orthogonally confirmed using ELISA. To gain insight into disease progression and severity we evaluated the altered protein expression with respect to clinically relevant phenotypic aspects: NPC Neurological Severity Score (NPC1 NSS), Annual Severity Increment Score (ASIS) and age of neurological onset.

**Results:**

This study identified multiple proteins with altered levels in CSF from individuals with NPC1 compared to non-NPC1 samples. These included proteins previously shown to be elevated in NPC1 (NEFL, MAPT, CHIT1, CALB1) and additional proteins confirmed by orthogonal assays (PARK7, CALB2/calretinin, CHI3L1/YKL-40, MIF, CCL18 and ENO2). Correlations with clinically relevant phenotypic parameters demonstrated moderate negative (*p* = 0.0210, *r* = -0.41) and possible moderate positive (*p* = 0.0631, *r* = 0.33) correlation of CSF CALB2 levels with age of neurological onset and ASIS, respectively. CSF CHI3L1 levels showed a moderate positive (*p* = 0.0183, *r* = 0.40) correlation with the concurrent NPC1 NSS. A strong negative correlation (*p* = 0.0016, *r* = -0.648) was observed between CSF CCL18 and age of neurological onset for childhood/adolescent cases. CSF CCL18 levels also showed a strong positive correlation (*p* = 0.0017, *r* = 0.61) with ASIS.

**Conclusion:**

Our study identified and validated multiple proteins in CSF from individuals with NPC1 that are candidates for further investigation in a larger cohort. These analytes may prove to be useful as supportive data in therapeutic trials.

**Trial registrations:**

NCT00344331, NCT00001721, NCT02931682.

**Supplementary Information:**

The online version contains supplementary material available at 10.1186/s40364-023-00448-x.

## Background

Niemann-Pick disease, type C1 (NPC1, MIM 257220) is a lethal, progressive, neurodegenerative disorder characterized by supranuclear vertical gaze palsy, cerebellar ataxia, cognitive impairment, and dementia [1, 2]. Typical onset is during childhood or adolescence, but adult cases are being more frequently recognized [3, 4]. The NPC1 phenotype is extremely heterogeneous and displays both a wide clinical spectrum of signs/symptoms and variable age of onset [2, 5, 6]. This is an ultrarare disorder with estimated incidence of the childhood/adolescent presentation on the order of 1/100,000, although an adult-onset variant may be more common [2, 7]. NPC1 is a recessive disorder most frequently due to pathological variants of *NPC1.* A similar, but less frequent cause of NPC is due to pathological variants of *NPC2* (MIM 607,625). Dysfunction of the NPC1 protein results in impaired transport of cholesterol out of the endolysosomal compartment, leading to accumulation of unesterified cholesterol [8] and glycosphingolipids [9, 10]. Various pathological processes may contribute to the clinical problems observed in NPC1 [11]. Lysosomal dysfunction and decreased cellular cholesterol bioavailability are likely the proximal causes that lead to mitochondrial dysfunction, oxidative stress, and calcium dysregulation among multiple aspects of the pathological cascade that contribute to neuronal dysfunction and loss. Recently, it has been shown that NPC1 also functions to regulate the activity of STING [12], thus providing a plausible explanation for the neuroinflammation that is a prominent aspect of NPC1 pathology [13–16].

Miglustat, a glycosphingolipid synthesis inhibitor, has shown efficacy in slowing neurological progression and increasing lifespan in individuals with NPC1 [17–20]. Although approved by the European Medicines Agency (EMA) and in most other countries, miglustat has not been approved for treatment of NPC by the US Food and Drug Administration (FDA). 2-hydroxypropyl-β-cyclodextrin (HPβCD) has shown significant therapeutic potential in both mouse [21–24] and cat [25] models of NPC1. Decreased neurological progression was also demonstrated at 18 months in a phase 1/2a trial of intrathecal HPβCD (VTS-270, adrabetadex) when compared to an age-appropriate natural history control cohort [26]. Arimoclomol, an HSP70 inducer, has also been reported to have preclinical efficacy in the NPC1 mouse model [27]. However, both a 12-month, sham-controlled, phase 2/3 trial of adrabetadex (NCT02534844) and a 12-month placebo-controlled trial of arimoclomol (NCT02612129, [28]) were unsuccessful, thus far, in obtaining regulatory approval. The rarity, slow clinical progression, and clinical heterogeneity of NPC1 all complicate the design and implementation of therapeutic trials.

The failure of both the arimoclomol and adrabetadex trials, in addition to failure of miglustat to obtain FDA approval, underscores the need for complementary data to support therapeutic efficacy. Biomarkers that can be used to monitor disease pathological activity and progression have the potential to provide supporting data, and pharmacodynamic biomarkers can be used to substantiate target engagement by an investigational drug. Prognostic biomarkers that provide insight into initiation of neurological progression may be useful in defining subgroups of trial candidates who are more likely to respond to therapy and will be clinically important in the future when considering initiation of treatment in neurologically asymptomatic infants identified by newborn screening [29]. In order to identify candidate protein biomarkers for NPC1 we compared protein levels in cerebrospinal fluid (CSF) samples from individuals with Niemann-Pick disease, type C1 and non-NPC1 controls using a multiplexed proximal extension assay. Multiplexed proximal extension assays have been used to characterize the CSF proteome in neurodegenerative disorders such as Alzheimer, Parkinson disease and multiple sclerosis [30, 31].

## Methods

### Trial participants and biomaterial collection

Individuals with NPC1 were enrolled in a natural history/observational trial being conducted at the National Institutes of Health Clinical Center (NCT00344331). This clinical study was initially approved by the *Eunice Kennedy Shriver* National Institute of Child Health and Human Development Institutional Review Board and ongoing review has been provided by the National Institutes of Health Intramural Institutional Review Board. Written consent for participation, and when appropriate, assent was obtained. Diagnosis of NPC1 was established by appropriate clinical, biochemical, or molecular testing. CSF was obtained by lumbar puncture and stored at -80 ºC. Ethical and statutory issues preclude obtaining CSF samples from healthy children. Rather than using adult control CSF we obtained age-appropriate CSF from three non-NPC1 cohorts. Anonymous pediatric control samples of known age and sex were obtained from CSF specimens that were originally collected for a clinical indication. Laboratory CSF samples with elevated cell count or positive culture were excluded. Additional comparison samples were obtained from ongoing natural history trials of Smith-Lemli-Opitz syndrome (SLOS, NCT00001721) and Creatine Transport Deficiency (CTD, NCT02931682). Unlike NPC1, neither SLOS nor CTD manifest neurodegeneration and these samples were collected and handled in a manner identical to the NPC1 samples. Both the SLOS and CTD protocols were approved by the *Eunice Kennedy Shriver* National Institute of Child Health and Human Development Institutional Review Board with ongoing review being provided by the National Institutes of Health Intramural Institutional Review Board. Written informed consent was obtained from guardians. NPC1 phenotype parameters included the 17-domain NPC Neurological Severity Score (NSS) [6], Annual Severity Increment Score (ASIS) [32] and age of neurological onset.  Sample identification, miglustat status, age and sex information is provided in Additional Table 1.  

### Proximal extension assay

Proximal Extension Assays (PEA, Olink® Explore 1536) were performed by Olink Proteomics, Inc. (Boston, MA). NPC1 and non-NPC1 control CSF samples (40 µl) were randomized on a 96-well plate, sealed, frozen and shipped overnight on dry ice to Olink. Normalized protein expression (NPX) values were provided by Olink and used as input to the differential abundance analysis.

### PEA statistical and bioinformatic analysis

All computational analysis described below was conducted using R version 4.0.

### Differential abundance analysis

The output of the Proximal Extension Assay consists of protein abundance measurements for proteins included in the targeted panels. Here the four panels used were Oncology, Cardiometabolic, Neurology, and Inflammation, with approximately 384 proteins in each panel. Prior to differential abundance analysis, protein abundance measurements across all panels were combined into a cleaned protein x sample matrix. In instances where a protein was measured in more than one panel, a suffix was added to the protein name, allowing both protein measurements to be present in the combined matrix. Protein abundance is represented as a normalized protein expression (NPX) value, which is on the log_2_ scale. Note that a cell in this matrix with an “NA” value indicates that there was an error in measuring the abundance of a given protein (row) in a given sample (column), or that protein abundance was too low to quantify. Any sample for which 75% or more of the NPX values were “NA” and any protein for which the NPX values was “NA” across 75% or more of samples were removed from the analysis. Using this approach, one NPC sample was removed because all proteins in the Cardiometabolic panel could not be measured. Additionally, principal component analysis (PCA) was performed and both sample and protein PCA plots were constructed to visually identify outliers (Additional Fig. 1). This approach identified two pediatric non-NPC1 samples as clear outliers that were removed in downstream analysis.

Differential protein abundance was then analyzed for various sample group comparisons. A Kruskal–Wallis rank-sum test was conducted for each protein to assess if protein abundance was statistically different between NPC1 and non-NPC1 samples. Kruskal–Wallis was selected for its robustness for data that is not normally distributed, and preliminary analysis showed that not all NPX values followed a normal distribution. *P*-values generated for each protein from the Kruskal–Wallis test were then adjusted to account for multiple testing using the Benjamini–Hochberg correction, also known as false discovery rate (FDR). Log_2_ fold change ratios between two conditions were calculated by taking the difference in median protein abundance between the two conditions, given that the NPX values are already on a log_2_ scale. Median, rather than mean, was used in this calculation to mimic the rank-based nature of Kruskal–Wallis. Proteins with an adjusted *p*-value (FDR) < 0.1 were identified as differentially abundant between the two conditions of the contrast. PCA, Kruskal–Wallis testing, and multiple testing correction were conducted with the R “stats” base package.

### Metadata covariate analysis

Since this study focuses on a neurodegenerative disorder, additional analyses were conducted to ensure that the results of the differential abundance analysis were not influenced by covariates that would obscure the true differences between, for example, NPC1 and non-NPC1 patients. Age and sex were assessed as potential covariates in the differential abundance analysis. To do so, the distribution of patient age was compared between sample groups in each contrast of interest, and a Wilcox rank-sum test was used to determine if the patient age distributions were significantly different between sample groups, indicating a potential covariate. Similarly, patient sex distributions were compared between sample groups for each contrast of interest, and a Chi-square test was used to assess statistical significance of distributional differences. For both metadata variables, there appeared to be distributional differences between NPC1 (Miglustat-untreated) and non-NPC1 samples, indicating that these variables may need to be included as covariates in the differential abundance analysis.

After identifying potential covariates, the PEA data were re-analyzed using an ANOVA model, implemented using the R “stats” base package, which allows for covariates to be introduced but assumes normally distributed data. An ANOVA model was constructed for each protein using Age as a covariate, and Benjamini–Hochberg was used for multiple testing correction. Comparing the adjusted p-values from the covariate ANOVA with those from Kruskal–Wallis, there were few differences in the set of proteins identified as differentially abundant, and so Kruskal–Wallis was selected as the appropriate model due to its robustness for non-normality.

### Metadata correlation analysis

This component of the project assessed whether trends in protein abundance correlate with prognostic metrics in NPC1 patients. A Spearman correlation coefficient was calculated for each protein, represented with Olink NPX abundance measurements, and metadata variable pair. The Spearman correlation analysis also produced a *p*-value for each protein-metadata variable pair, which was then corrected using Benjamini–Hochberg to account for multiple testing. Correlations between a protein and metadata variable were considered statistically significant for adjusted *p*-value < 0.1.

### Validation assays

Human specific enzyme-linked immunosorbent assays (ELISA) were used to validate CCL18, FABP5, PARK7, CKMT1, INPP1, SNCG, CHI3L1, MIF, SCRN1 and CALB2. Samples were thawed on ice and assayed with specific ELISAs as outlined by the manufacturers as follows: CCL18, FABP5 and PARK7 (Abcam, Waltham, MA, USA); CKMT1, INPP1 and SNCG (MyBIOSource, San Diego, CA, USA); CHI3L1 (Quidel Corporation, San Diego, CA, USA), MIF (R&D Systems, Minneapolis, MN, USA); SCRN1 (Abbexa LLC, Sugar Land, TX, USA) and CALB2 (Cloud-Clone Corporation, Houston, TX, USA). CSF was diluted appropriately with sample diluent and assayed in duplicate or singlets. Single determinations were performed where there was a limited amount of CSF. There was a strong correlation (*r* = 0.93) between the NPX predicted fold-change and the ELISA fold-change for PARK7, CALB2, CHI3L1, MIF and CCL18. All values for CKMT1, INPP1, SCRN1 and SNCG were below the limit of detection. ENO2 levels and corresponding normal values were obtained from Mayo Clinic Laboratories (https://www.mayocliniclabs.com/test-catalog/overview/81796).

### Statistical analysis and graphing

Graphs were generated using R with the ggplot2 package or GraphPad Prism 9.4.0. For validation by ELISA, non-NPC1 and NPC1 results were evaluated using unpaired, two-tailed t-test. Individual values that were below the limit of detection were set at the limit of detection. Pearson correlations were used to evaluate log_10_ transformed analyte concentrations and clinical parameters. Spearman correlations with *r*-values of 0.1–0.3, 0.3–0.5 or > 0.5 were considered weak, moderate, or strong, respectively.

## Results

### Demographic and clinical characterization of the NPC1 and non-NPC1 control cohorts

Our study population consisted of 28 individuals with NPC1 and 30 non-NPC1 comparison samples. Fourteen of the individuals with NPC1 had been treated with miglustat. Ethical considerations do not allow for the collection of CSF from healthy children. Pediatric comparison samples were acquired from three sources. Pediatric laboratory control (PLC) CSF (*n* = 8) was obtained from residual clinical samples. Prior to obtaining these samples, we had no control over handling or storage time, and two additional pediatric non-NPC1 samples identified as clear outliers were removed from analysis. To control for handling/processing issues, we also assayed CSF samples from individuals with either SLOS (*n* = 10) or CTD (*n* = 12) which were collected, processed, and stored in a manner identical to the NPC1 samples. Although SLOS and CTD are neurodevelopmental disorders, unlike NPC1, neurodegeneration is not observed in either SLOS or CTD. Demographic information for the NPC1 and non-NPC1 comparison cohorts is provided in Table 1. Due to the inclusion of some adult cases, the average age of the NPC1 cohort (17.1 ± 18.3 years) was greater, but not significantly, than the non-NPC1 controls (9.5 ± 5.9 years, *p* = 0.24), but median age was similar (11.40 and 8.0 years, respectively). 54% of the NPC1 participants were male, whereas 69% of the non-NPC1 controls were male. CTD is an X-linked disorder and thus contributed to the preponderance of males in the non-NPC1 group. Although survival time is increased in female *Npc*^*null/null*^ mice, this sex difference was not observed in individuals with NPC1 [33]. Clinical characteristics of the NPC1 cohort are provided in Table 2. Mean NPC Neurological Severity Score (NSS), Annual Severity Increment Score (ASIS) and age of neurological onset were 16.8 ± 11.0 points, 1.95 ± 2.26 points/year, and 8.47 ± 12.02 years, respectively. Although no *p*-values were < 0.05, the miglustat treated cohort had lower mean NPC NSS (*p* = 0.07), ASIS (*p* = 0.61) and age of neurological onset (*p* = 0.23) (Table 2).

**Table 1:** Demographic characterization of comparison and NPC1 cohorts

Cohort	n	Mean Age (years)	Median Age and Range (years)	Male/Female (%)
Controls	30	9.5 ± 5.9	8.0 (0.4–21.0)	21/9 (70%)
PLC	8	10.6 ± 7.7	11.0 (0.4–20.0)	4/4(50%)
SLOS	10	10.5 ± 6.4	9.4 (2.6–21)	5/5 (50%)
CTD	12	7.8 ± 4.0	7.0 (3.2–14.3)	12/0 (100%)
NPC1	28	17.1 ± 18.3*	11.4 (0.9–68)	15/13 (54%)

^*^Mann Whitney *p* = 0.24 Control versus NPC1

**Table 2:** Clinical characteristics of the NPC1 cohort

	**n**	**Mean**	*p*-value*	**Median (range)**
NPC NSS (points)	28	16.8 ± 11.0	-	18.0 (0–42)
No Miglustat	14	20.0 ± 8.2		20.0 (0–32)
Miglustat	14	13.6 ± 12.8	0.07	13.6 (0–42)
ASIS (points/year)	26**	1.95 ± 2.26	-	1.07 (0.31–9.37)
No Miglustat	13	2.25 ± 2.69		1.31 (0.31–9.37)
Miglustat	13	1.64 ± 1.80	0.61	0.89 (0.31–6.09)
Age of Neurological Onset (years)	28	8.47 ± 12.02	-	3.5 (1.3–43)
No Miglustat	14	12.07 ± 15.79		5.0 (1.5–43)
Miglustat	14	4.86 ± 4.79	0.23	2.0 (1.3–16)

^*^Mann–Whitney t-test

^**^2 individuals with NPC NSS = 0

### Identification of proteins with altered levels in cerebrospinal fluid

**Fig. 1:**
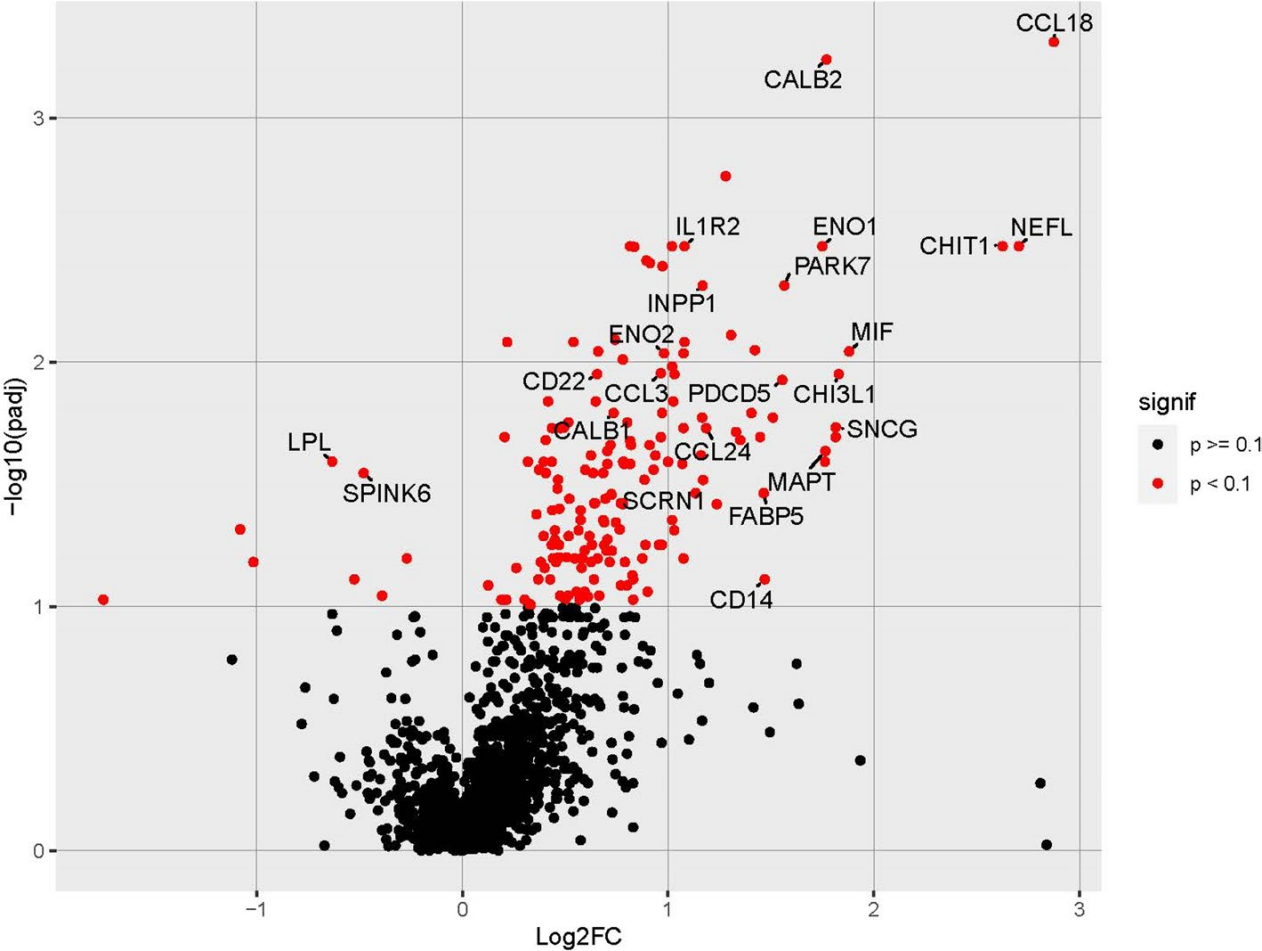
Volcano plot of differentially expressed proteins comparing NPC1 and non-NPC1 cerebrospinal fluid samples. Elevated log2(FC) values indicate increased protein expression in CSF from individuals with NPC1. 177 proteins with adjusted *p*-value of < 0.10 are indicated in red. Proteins discussed in the manuscript are labeled

Relative abundance of CSF proteins was determined by Proximal Extension Assay (PEA, Olink Explore 1536). Data was obtained on 1467 proteins (additional Tables 2 and 3). Pairwise comparison between the three non-NPC1 groups (CTD vs PLC, SLOS vs PLC and CTD vs SLOS) and the 14 non-miglustat NPC1 samples did not identify any proteins within the comparison groups with differential levels (FDR < 0.10). Thus, the three non-NPC1 sets were combined and compared to the NPC1 cohort not on miglustat at the time of sample collection. We identified 177 proteins with altered levels in NPC1 CSF (additional Table 2, columns C and D). A volcano plot is shown in Fig. 1. Increased levels were observed for 169 proteins and decreased levels were observed for eight. Table 3 provides a list of the top 50 proteins with increased levels ranked by fold change, and Table 4 provides a list of the 8 proteins with decreased levels. We were unable to identify any proteins with an adjusted *p*-value < 0.10 when comparing the no miglustat and miglustat treated cohort. When the two NPC1 cohorts (± miglustat)were combined, we identified 286 proteins with increased levels and 9 with decreased levels (additional Table 2, columns D and E). The top 50 proteins with significantly increased expression, ranked by fold-change, identified by these two analyses were very similar. Only five additional proteins (CKMT1A_CKMT1B, SIGLEC5, CLPS, MAFP5, and ACP5) were identified as being significantly (*p* < 0.10) elevated when the two NPC1 cohorts were combined (additional Table 2). All of the top 50 proteins, ranked by fold-change, identified when using the non-miglustat NPC1 sample set were significantly elevated in the combined cohort analysis.

**Table 3:** Top 50 proteins with increased expression in NPC1 CSF

Gene	Protein	adj. *p*-value	Fold Change
*CCL18*	C–C Motif Chemokine Ligand 18	4.88E-04	7.36
*NEFL*	Neurofilament light	3.36E-03	6.50
*CHIT1*	Chitotriosidase-1	3.36E-03	6.19
*MIF*	Macrophage Migration Inhibitory Factor	9.05E-03	3.68
*CHI3L1*	Chitinase-3-like protein 1, YKL-40	1.12E-02	3.53
*SNCG*	Gamma-synuclein	1.85E-02	3.53
*TMSB10*	Thymosin β-10	2.03E-02	3.51
*CALB2*	Calretinin	5.77E-04	3.41
*CPVL*	Carboxypeptidase Vitellogenic Like	2.56E-02	3.41
*MAPT*	Microtuble Associated Protein Tau	2.32E-02	3.39
*ENO1*	Enolase 1	3.36E-03	3.36
*PARK7*	Parkinson Associated Deglycanase, DJ-1	4.87E-03	2.97
*PDCD5*	Programmed Cell Death Protein 5	1.19E-02	2.95
*DNAJA2*	DnaJ Heat Shock Protein Family (Hsp40) member A2	1.69E-02	2.85
*FABP5*	Fatty Acid Binding Protein 5	3.44E-02	2.77
*CD14*	Cluster of Differentiation 14	7.74E-02	2.75
*NSFL1C*	NSFL1 cofactor p47	2.03E-02	2.73
*RBKS*	Ribokinase	8.95E-03	2.68
*ATOX1*	Antioxidant 1 Copper Chaperone	1.62E-02	2.64
*GLO1*	Lactoylglutathione lyase	2.09E-02	2.55
*LILRB2*	Leukocyte Immunoglobulin Like Receptor A2	1.94E-02	2.51
*NOS1*	Nitric oxide synthase 1 (neuronal)	7.76E-03	2.48
*WARS*	Tryptophanyl-tRNA synthetase, cytoplasmic	1.73E-03	2.45
*GDF15*	Growth Differentiation Factor 15	3.81E-02	2.36
*CCL24*	C–C Motif Chemokine Ligand 24	1.87E-02	2.27
*INPP1*	Inositol Polyphophate 1-phosphatase	4.87E-03	2.25
*ITGB2*	Integrin beta chain-2	3.05E-02	2.25
*ATP5IF1*	ATP Synthase Inhibitory factor Subunit 1	1.69E-02	2.23
*LILRA2*	Leukocyte immunoglobulin-like receptor subfamily A member 2	2.42E-02	2.23
*SCRN1*	Secernin-1	3.44E-02	2.19
*IL1R2*	Interleukin 1 Receptor Type 2	3.36E-03	2.11
*DDC*	DOPA Decarboxylase	8.29E-03	2.11
*GLOD4*	Glyoxalase domain-containing protein 4	9.22E-03	2.11
*PEBP1*	Pohosphatidylethanolamine-binding protein	1.87E-02	2.11
*MSR1*	Macrophage scavenger receptor 1	2.62E-02	2.10
*CPPED1*	Calcineurin-like phosphoesterase domain-containing protein 1	6.36E-02	2.10
*PPP3R1*	Calcineurin subunit B type 1	1.12E-02	2.04
*CCL8*	Chemokine (C–C motif) ligand 8	1.45E-02	2.04
*KLK4*	Kallikrein-related peptidase 4	4.88E-02	2.04
*PTS*	6-pyruvoyl-tetrahydropterin synthase	3.36E-03	2.03
*PHOSPHO1*	Phosphoethanolamine/phosphocholine phosphatase 1	1.04E-02	2.03
*TXNRD1*	Thioredoxin Reductase 1	4.42E-02	2.03
*CD300LF*	CMRF35-like molecule 1	2.56E-02	2.00
*ENO2*	Neuron specific enolase	9.22E-03	1.97
*OLR1*	Oxidized low density lipoprotein receptor 1	5.60E-02	1.96
*LGALS8*	Gelectin 8	1.62E-02	1.96
*SCGN*	Secretagogin	4.05E-03	1.96
*CCL3*	Macrophage Inflammatory Protein 1-alpha	1.11E-02	1.96
*LGMN*	Legumain	2.03E-02	1.94

**Table 4:** Proteins with decreased expression in NPC1 CSF

Gene	Protein	adj. *p*-value	Fold-change
*CLEC4C*	C-type lectin domain family 4 member C, CD303	6.36E-02	0.83
*LHB*	Luteinizing hormone, beta polypeptide	9.04E-02	0.76
*SPINK6*	Serine protease inhibitor Kazai-type 6	2.85E-02	0.72
*PDGFRA*	Platelet derived growth factor receptor alpha	7.74E-02	0.69
*LPL*	Lipoprotein lipase	2.56E-02	0.65
*CD302*	C-type lectin domain family 13 member A	6.58E-02	0.49
*CCL5*	Chemokine (C–C motif) ligand 5	4.84E-02	0.47
*AGR2*	Protein disulphide isomerase family member	9.38E-02	0.30

### Validation of differentially expressed proteins identified by proximal extension assay

Several of the top 50 proteins (Table 3) have previously been reported to be elevated in CSF from individuals with NPC1. These include NEFL [34, 35], MAPT [36] and CCL3 [13]. Although not in the top 50, CSF CALB1 levels were significantly elevated (*p* = 0.0162, FC = 1.67). CALB1, a marker of Purkinje neuron damage, was previously shown to be elevated in CSF from both individuals with NPC1 and the NPC1 cat model [37]. Similarly, CD22 CSF levels were increased (*p* = 0.0112, FC = 1.57). We have previously shown that CD22, a modulator of microglial function [38], is increased in CSF from individuals with NPC1 [14]. NEFL, CHIT1 and CCL18 have previously been shown to be elevated in plasma from individuals with NPC [39–41]. Identification of these proteins in this study supports the validity of this approach to identify proteins whose expression is altered in CSF from individuals with NPC1.

**Fig. 2:**
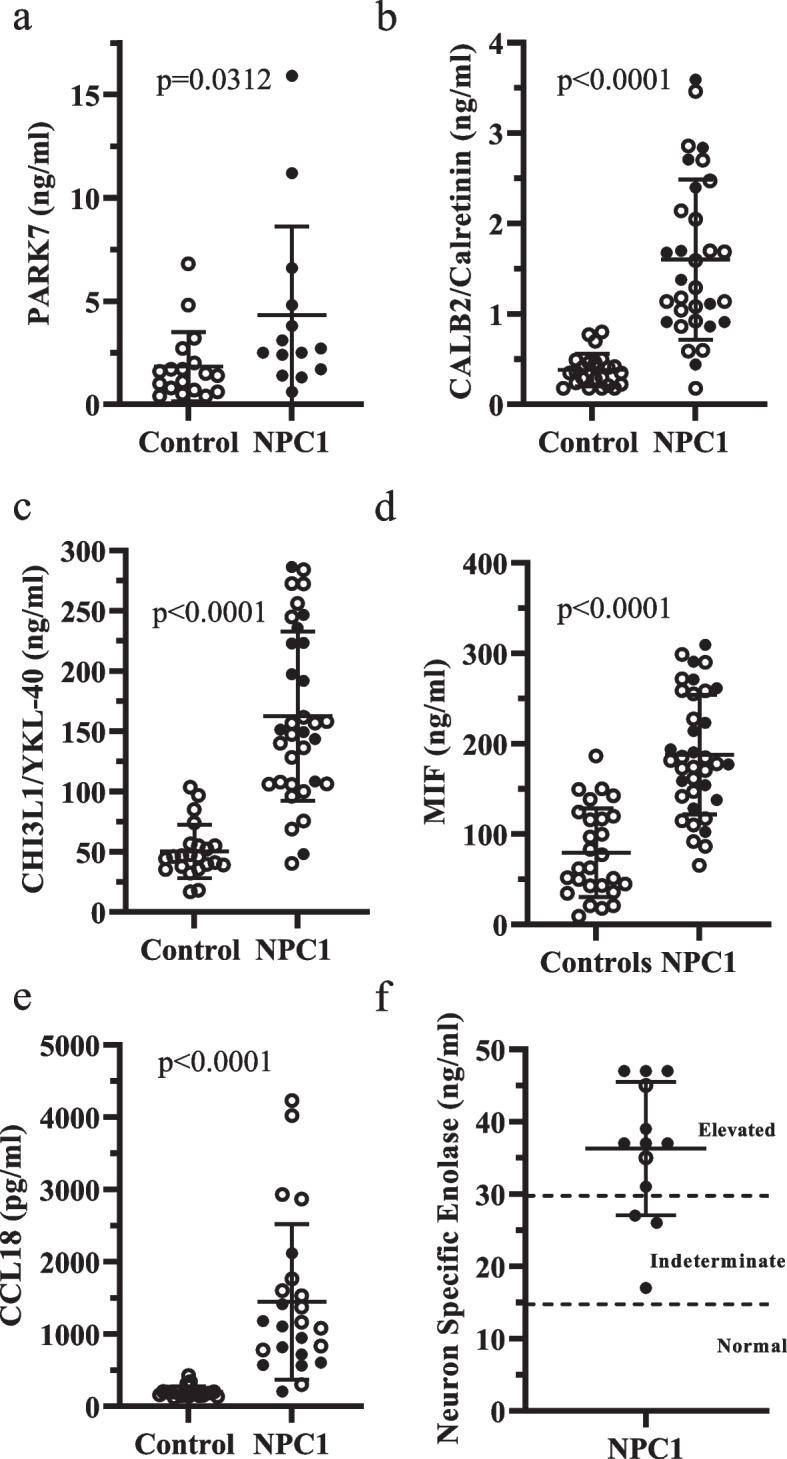
Validation of differentially expressed proteins. ELISA assays for PARK7 (**a**), CALB2/calretinin (**b**), CHI3L1/YKL-40 (**c**), MIF (**d**) and CCL18 (**e**) confirmed increased levels of these five proteins in cerebrospinal fluid from individuals with NPC1 relative to age-appropriate comparison samples. Laboratory testing also validated increased levels of neuron specific enolase (ENO2) in cerebrospinal fluid from individuals with NPC1 (**f**). Samples obtained from individuals with NPC1 on miglustat are indicated by an open circle. *P*-values are from unpaired, two-sided t-tests

To further validate the discovery PEA data, we evaluated selected proteins by ELISA. Using these orthogonal assays, we confirmed increased expression of PARK7, CALB2 (calretinin), CHI3L1/YKL-40, MIF and CCL18 (Fig. 2a-e). Although the range of values was larger for NPC1 CSF FABP5 levels (additional Fig. 2), they could not adequately be distinguished (*p* = 0.25) from non-NPC1 samples.

 Mean PARK7 levels (Fig. 2a) were elevated ~ 2.4-fold in NPC1 CSF (4.32 ± 4.30 ng/ml) relative to non-NPC1 comparison samples (1.83 ± 1.67 ng/ml, *p* = 0.0312). In contrast, NPC1 CSF CALB2 (Fig. 2b, 1.60 ± 0.89 ng/ml, *p* < 0.0001) and CHI3L1/YKL-40 (Fig. 2c, 162.6 ± 70.3 ng/ml, *p* < 0.0001) levels were both markedly elevated relative to age-appropriate non-NPC1 samples (0.38 ± 0.18 ng/ml and 50.28 ± 22.20 ng/ml, respectively). CALB2 levels were elevated ~ 4.2-fold and CHI3L1 levels were elevated ~ 3.2-fold. Both CHI3L1 (40.3–286.4 ng/ml) and especially CALB2 (0.18–3.59 ng/ml) manifested a relatively large dynamic range of approximately 7- and 20-fold, respectively. Macrophage migration inhibitory factor (MIF) NPC1 CSF levels (Fig. 2d) were elevated (188 ± 66 ng/ml) ~ 2.4-fold above non-NPC levels (80 ± 49 ng/ml, *p* < 0.0001) with a dynamic range of ~ 3.7-fold (65–310 ng/ml). NPC1 CSF CCL18 levels (Fig. 2e) were markedly elevated (1.45 ± 1.08 pg/ml) ~ 7.3-fold relative to non-NPC1 control levels (0.20 ± 0.08 ng/ml, *p* < 0.0001) and demonstrated a large dynamic range (20-fold, 0.21–4.23 ng/ml). Increased CSF levels of ENO2 (neuron specific enolase) were confirmed by clinical laboratory testing. CSF ENO2 levels were elevated in individuals with NPC1 relative to the normal clinical laboratory range (Fig. 2f). Mean ENO2 level in thirteen NPC1 samples was 36.3 ± 9.7 ng/ml. Normal CSF ENO2 levels reported by Mayo Clinical Laboratories are considered ≤ 15 ng/ml and elevated CSF levels are considered ≥ 30 ng/ml. This normal reference cut-off value is consistent with < 12.0 ng/ml that was reported by Kay et al. [42] for pediatric (< 15 years old) samples. Of the thirteen NPC1 samples, 10 were elevated and 3 were indeterminate (> 15 but < 30 ng/ml). Taken together the identification of proteins previously reported to be elevated in NPC1 as described above and orthogonal confirmation of PARK7, CALB2, CHI3L1/YKL-40, MIF, CCL18 and ENO2 strongly supports the validity of proteins identified by the PEA screen.

The effect of miglustat therapy on CSF protein biomarkers was explored. No change in CALB2, CHI3L1, MIF or FABP5 levels was observed when comparing NPC1 samples from NPC1 individuals not on miglustat and individuals on miglustat (additional Fig. 3). We did observe increased levels of CCL18 in NPC1 samples from individuals on miglustat (1884 ± 1246 ng/ml) relative to individuals not on miglustat (932 ± 518 ng/ml, *p* = 0.0275) (additional Fig. 3). This was not anticipated and may simply be a consequence of the relatively small number of samples tested. Miglustat therapy is known to alter the microglial phenotype in the feline model [43] and this observation may provide some insight into the role of CCL18 in regulation of the neuroinflammatory response in NPC1. However, this result needs to be confirmed in a larger sample set.

### Phenotype correlations with proximal extension assay NPX values

To explore whether our PEA data provided insight into potential biomarkers that correlated with phenotypic aspects of NPC1, we evaluated the relationship between PEA NPX values for 1467 proteins and the clinical outcome parameters of age of neurological onset, NPC NSS and ASIS. Identification of these correlations with NPX values may be useful in prioritizing protein analytes to investigate further. Given the wide range of age included in the NPC1 cohort, we also explored the potential relationship between age and protein expression to determine if this could be a confounder. Correlations with adjusted *p* < 0.10 were observed between age and NPX values for 34 proteins (additional Table 4). Given this effect of age on many proteins, we explored whether accounting for age would impact the potential biomarker list. For this we used an ANOVA model with age as a covariate. The ANOVA model identified 182 differentially expressed proteins in comparison to the 177 identified using Kruskal–Wallis test with no covariates. Among the top 50, based on fold-change, only one additional protein, CLEC5A (*p* = 0.009, FC = 2.06) was identified.

**Fig. 3:**
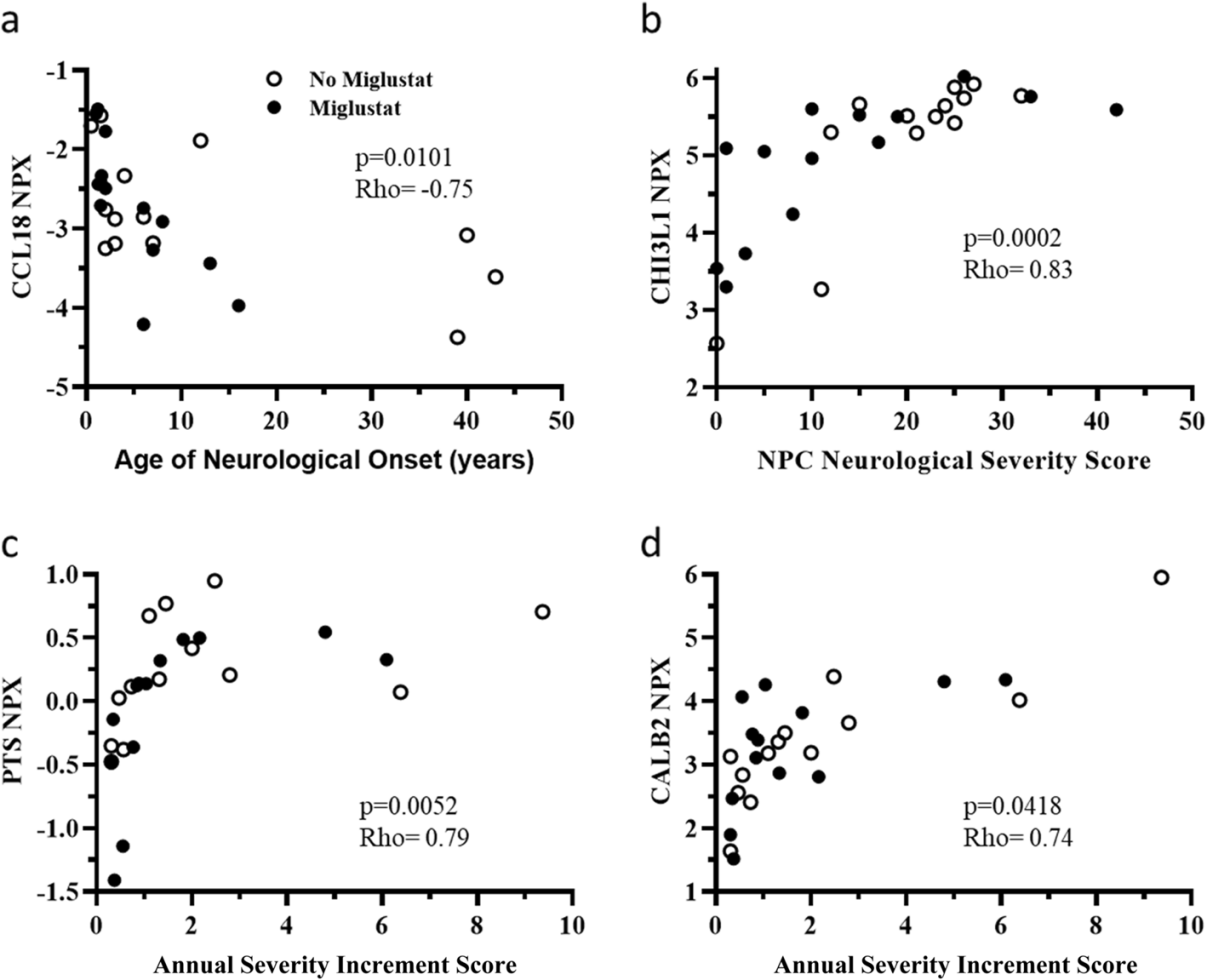
Clinical phenotype correlations with Proximal Extension Assay NPX values. **a** CSF CCL18 NPX values showed a negative correlation (*p* = 0.0101, rho = -0.75) with age of neurological onset. Open circles represent individuals not on miglustat and filled circles represent individuals on miglustat. NPX correlations were performed on the combined cohorts. **b** CSF CHI3L1 NPX values were positively correlated ( *p* = 0.0002, rho = 0.83) with the total NPC Neurological Severity Score. Both CSF 6-pyruvoyl-tetrahydropterin-synthase (PTS) (**c**) and CALB2 (**d**) NPX values were positively correlated (*p* = 0.0052, rho = 0.79 and *p* = 0.0418, rho = 0.74, respectively) with Annual Severity Increment Scores. Y-axis NPX values are log_2_

Correlation of NPX values with age of neurological onset identified strong negative correlations with IL7 (*p* = 0.0015, rho = -0.78, data not shown) and CCL18 (*p* = 0.0101, rho = -0.75,). IL7 NPX values also correlated with age (*p* = 0.0036, rho = -0.79, data not shown) and this likely confounds interpretation of the correlation with age of neurological onset. The correlation between CCL18 and neurological age of onset is shown in Fig. 3a. CHI3L1 NPX values showed a strong positive correlation (*p* = 0.0002, rho = 0.83) with concurrent NPC NSSs (Fig. 3b). Both 6-pyruvoyl-tetrahydropterin-synthase (PTS, *p* = 0.0052, rho = 0.79) and CALB2 (*p* = 0.0418, rho = 0.74) had strong positive correlations with ASIS values (Fig. 3c,d). No apparent differences were noted when comparing individuals untreated or treated with miglustat (Fig. 3).

### Phenotype correlation with ELISA quantification data

Increased expression of CCL18, MIF, CALB2 and CHI3L1 in the PEA was validated by analyte specific ELISA (Fig. 2). CCL18, CALB2 and CHI3L1 all demonstrated relatively wide dynamic ranges and PEA NPX values correlated significantly with different aspects of the NPC phenotype (Fig. 3). To further investigate and confirm their potential as clinically relevant biomarkers, we investigated whether the CSF levels of these proteins in the validation ELISA assays correlated with Age of Neurological Onset, total NPC NSS or ASIS.

**Fig. 4:**
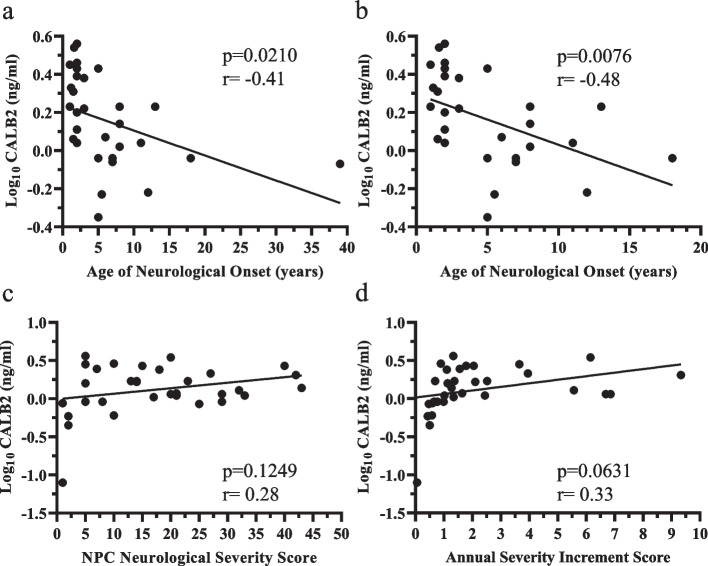
Correlation of NPC1 cerebrospinal fluid CALB2/Calretinin levels with NPC1 clinical phenotype. **a** Decreased CSF CALB2 levels were associated with later age of neurological disease onset in individuals with NPC1 (*p* =  = 0.0210, rho = -0.41). **b** The negative correlation between CSF CALB2 levels and age of neurological onset (*p* = 0.0076, rho = -0.48) was also present when excluding a 40 yo individual. CSF CALB2 levels did not correlate with the concurrent NPC Neurological Severity Score (**c**). CALB2 levels showed a modest correlation with the Annual Severity Increment Scores (**d**)

CSF CALB2 levels had a moderate negative correlation with age of neurological onset (Fig. 4a, *p* = 0.0210, rho = -0.41). This moderate negative correlation remained (Fig. 4b, *p* = 0.0076, *r* = -0.48) when age of neurological onset was restricted to classical childhood/adolescent onset (< 20 years old). Consistent with the CALB2 NPX data, CSF CALB2 levels did not correlate with NPC NSS but showed a moderate correlation (*p* = 0.0631, *r* = 0.33) with ASIS (Fig. 4c,d).

**Fig. 5:**
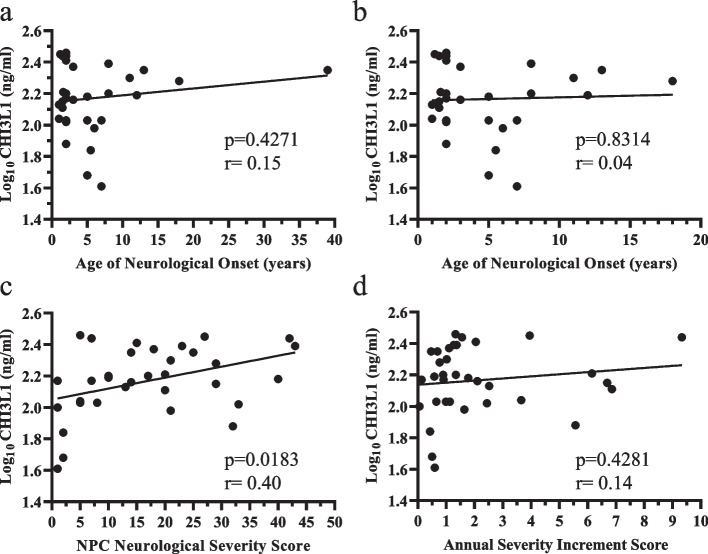
Correlation of NPC1 cerebrospinal fluid CHI3L1 levels with NPC1 clinical phenotype. CSF CHI3L1 levels did not correlate with either age of neurological onset (**a**, **b**) or Annual Severity Increment Scores (**d**). However, CSF CHI3L1 levels showed a positive correlation (*p* = 0.0183, rho = 0.40) with the concurrent NPC Neurological Severity Scores (**c**)

**Fig. 6:**
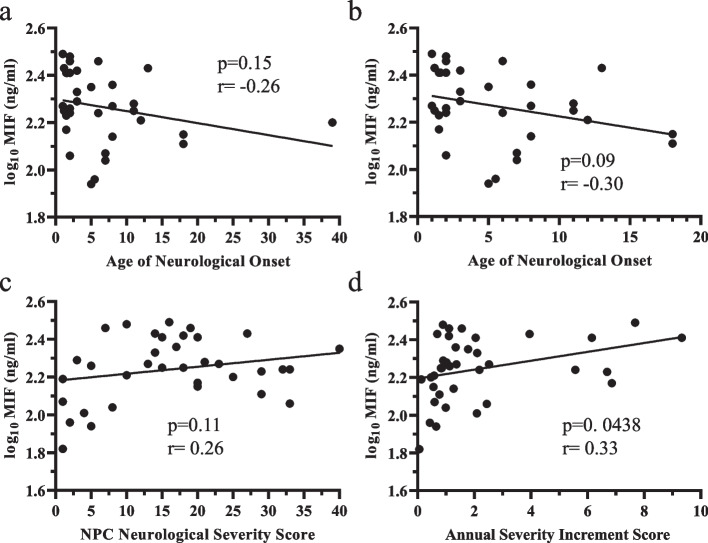
Correlation of NPC1 cerebrospinal fluid Macrophage Migration Inhibitory Factor (MIF) levels with NPC1 clinical phenotype. CSF MIF levels did not correlate with either age of neurological onset (**a**, **b**) or the concurrent NPC Neurological Severity Score (**c**). There was a modest correlation of increased CSF MIF levels with increased Annual Severity Increment Scores (**d**)

Neither CSF CHI3L1 nor MIF demonstrated good correlations with phenotypic findings. CSF CHI3L1 levels did not correlate with either age of neurological onset or ASIS but had a moderate positive correlation (*p* = 0.0183, *r* = 0.40) with concurrent NPC NSS (Fig. 5). CSF MIF levels did not correlate with age of neurological onset or NPC NSS. There was a marginally moderate positive correlation (*p* = 0.0438, *r* = 0.33) between CSF MIF levels and ASIS (Fig. 6). Of the four proteins for which we performed these analyses, CHI3L1 and MIF had the smallest dynamic ranges.

**Fig. 7:**
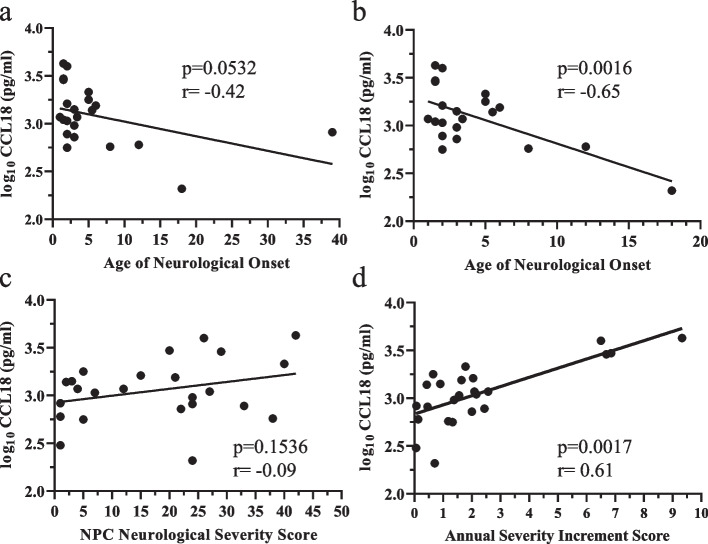
Correlation of NPC1 cerebrospinal fluid CCL18 levels with NPC1 clinical phenotype. There appeared to be a possible negative correlation (*p* = 0.0532, rho = -0.42) between CSF CCL18 levels and Age of Neurological Onset when evaluating the entire cohort (**a**); however, the negative correlation became readily apparent (*p* = 0.0016, rho = -0.65) when evaluating individuals with an age of neurological onset < 20 years (**b**). **c**) We did not observe a correlation between CSF CCL18 levels and the concurrent NPC Neurological Severity Score. **d**) we did observe a strong positive correlation (*p* = 0.0017, rho = 0.61) between CSF CCL18 levels and Annualized Severity Index Scores

CSF CCL18 levels, assessed by ELISA, show a moderate negative (*p* = 0.0532, rho = -0.42) correlation with age of neurological onset (Fig. 7a). When limited to classical childhood/adolescent disease with age of neurological onset less than 20 years old, a strong negative correlation (Fig. 7b, p = 0.0016, *r* = -0.648) was observed. This conclusion is consistent with the negative correlation between CCL18 NPX levels and age of neurological onset observed in the PEA data (Fig. 3a). We did not observe a significant correlation of CCL18 levels with the concurrent NPC NSS (Fig. 7c); however, we did observe a strong positive correlation (*p* = 0.0017, *r* = 0.61 between CSF CCL18 levels and ASIS (Fig. 7d).

## Discussion

Niemann-Pick disease type C1 (NPC1) is a rare, lethal, neurodegenerative disease without adequate therapy. Although recognition of adult cases is becoming more common, NPC1 is primarily a disease of children/adolescents. There is a critical need to develop and test potential therapeutic interventions. Developing therapies for NPC1 is greatly complicated by the rarity of the disease combined with marked phenotypic heterogeneity and progression that occurs over years. This difficulty is underscored by the recent failure of controlled 12-month therapeutic trials of intrathecal 2-hydroxypropyl-β-cyclodextrin (VTS-270/adrabetadex, NCT02534844) and oral arimoclomol (NCT02612129) to be approved by the FDA even though both showed clinical promise [26, 28]. Similarly, miglustat, although approved by the EMA, failed to gain approval by the FDA based on a 12-month trial. However, miglustat has been shown, under real-life conditions, to have clinical efficacy [17, 19, 20, 44]. However, establishing efficacy for a slowly progressing, rare and heterogeneous diseases such as NPC1 takes years. The disease pathology is not conducive to demonstrating therapeutic efficacy in short-term classical placebo-controlled trials. The likely need to develop multidrug therapy targeting different aspects of the NPC1 pathological cascade only compounds the difficulty of developing an effective therapeutic approach.

Biomarkers are tools that can be used to facilitate clinical care and drug development. Clinically they can be used to facilitate diagnosis and as prognostic indicators. They can be used in drug development to identify pathological processes that may be amenable to therapy, as pharmacodynamic measures and as surrogates for, or reasonable indicators of, clinical response.

### Lipid, oxysterol and bile acid diagnostic and pharmacokinetic biomarkers in NPC1

A number of blood-based diagnostic biomarkers have been developed for NPC1. These include 3β,5α,6β-cholestane-triol [45, 46] and N-palmitoyl-O-phosphocholineserine (PPCS, lyso-509) [47–49]. N-(3β,5α,6β-trihydroxy-cholan-24-oyl)glycine, a glycinated bile acid metabolite of 3β,5α,6β-cholestane-triol can also be used for diagnosis [50] and has shown particular utility as an analyte for newborn screening [51, 52]. Prior to development of these blood-based tests, laboratory diagnosis required a skin biopsy combined with assessment of unesterified cholesterol storage by filipin staining [53] or molecular testing. The difficulty in establishing a diagnosis of NPC prior to establishment of the blood-based tests likely contributed to a significant diagnostic delay of 4–5 years [6]. In addition to their utility as diagnostic analytes, oxysterols, specifically 24(S)-hydroxycholesterol, has shown utility as a pharmacodynamic biomarker [54]. 24(S)-hydroxycholesterol is an enzymatically produced biomarker of central nervous system neuronal cholesterol homeostasis. 24(S)-hydroxycholesterol is decreased in individuals with NPC1 [46] and has been used to monitor restoration of cholesterol homeostasis in both preclinical models [54] and humans with NPC1 [26, 54–56].

### Previously identified CSF and serum/plasma protein biomarkers in NPC

Several CSF proteins have been reported to be elevated in individuals with NPC1 and may have utility as therapeutic response biomarkers. CSF calbindin D (CALB1), fatty acid binding protein 3 (FABP3), tau (MAPT), amyloid peptide (Aβ_40-42_) and neurofilament light (NEFL) levels have been shown to decrease in response to miglustat therapy [34, 36, 37, 57, 58]. CALB1, FABP3, MAPT and NEFL are biomarkers of neuronal damage. CALB1 is of particular interest in NPC1 since it is highly expressed in cerebellar Purkinje neurons, the loss of which underlie the cerebellar ataxia which is a predominant symptom in NPC1. CSF CALB1 and FABP3 levels have been shown to decrease in response to administration of intrathecal 2-hydroxypropyl-β-cyclodextrin [26]. Elevated CSF levels of interleukin 3, chemokine ligand 5, interleukin 16, Mip1α (CCL3), and CD22 are indicative of the neuroinflammation observed in NPC1, and CD22 levels may decrease in response to intrathecal administration of HPβCD [13, 14, 59]. Oyama et al. [60] reported decreased CSF hypocretin-1 levels in a case series of individuals with NPC1. Altered hypocretin-1 levels may contribute to gelastic cataplexy, a nearly pathognomonic sign of NPC1 in children [5, 61]. Except for NEFL [34], data on these CSF biomarkers is limited to a relatively small number of samples.

Multiple serum/plasma proteins have been shown to be elevated in individuals with NPC1. These include GPNMB, LGAL3, cathepsin D, cathepsin S and lysozyme [62–66]. It is not clear to what degree serum/plasma elevations of these proteins are reflecting peripheral versus central nervous system disease. Increased serum NEFL levels have been reported in small numbers of individuals with NPC1 [35, 39, 56]. NEFL is a marker of neuronal damage and central nervous system derived NEFL, although at significantly lower concentrations, is detectable in serum [67]. Again, data on these potential blood-based protein biomarkers is limited. Analysis of a much larger cohort of samples will be necessary to determine their utility as tools to support clinical assessment or drug development.

### Identification of novel CSF protein biomarkers with increased levels in NPC

The goal of this current project was to both confirm and extend our knowledge of CSF proteins that may have utility as biomarkers for NPC1. Identification of disease relevant biomarkers can provide tools to facilitate clinical care or support drug development. This study identified several proteins which have previously been reported to be elevated in CSF from individuals with NPC1. These include NEFL [34], MAPT [36], CALB1 [37], CD22 [14], and CCL3 [13]. In addition, increased levels of CHIT1 and CCL18 in plasma from individuals with NPC1 have also been observed [40]. In addition to relying on previous studies, we used orthogonal assays to validate our PEA results. ELISA assays validated significantly elevated CSF levels for PARK7 (*p* = 0.0312), CALB2/calretinin (*p* < 0.0001), CHI3L1/YKL-40 (*p* < 0.0001), MIF (*p* < 0.0001) and CCL18 (*p* < 0.0001). To our knowledge this is the first demonstration that these five protein analytes are elevated in NPC1 CSF. Although the mean level was increased, we were not able to confirm an increased level of FABP5 (*p* = 0.11) in CSF from individuals with NPC1. ENO2 levels in NPC1 CSF were consistently elevated relative to clinical laboratory normal levels. These orthogonal validations combined with identification of proteins which had previously been shown to be elevated in NPC1 CSF strongly supports the conclusion that the PEA screen was successful in accurately identifying proteins with altered expression. Although each specific protein will need to be validated, these data do provide a list of candidate biomarkers that can be evaluated to determine if they correlate with clinical phenotype and respond to therapeutic interventions.

ENO2, neuronal specific enolase or enolase γ, is a glycolytic enzyme expressed by neurons and neuroendocrine cells. Elevated levels of ENO2 are observed in neurodegenerative diseases and increased levels correlate with disease progression (reviewed in [68]). ENO2 is considered a marker of neuronal damage, but it may also play a direct role in promoting neuroinflammation. Archived frozen samples can be used to assess NSE levels [69], thus ENO2 is a candidate for future studies focused on evaluating its utility as a biomarker in NPC1.

Pathological variants of PARK7 (DJ-1) have been reported in individuals with early onset Parkinson disease [70]. PARK7 is a redox-sensitive deglycase which inhibits the aggregation of α-synuclein and protects neurons against oxidative stress. Recently it has been shown that PARK7 prevents protein damage by 1,3-bisphosphoglycerate, a glycolytic metabolite [71]. Altered glycolysis and glycolytic enzymes have been described in NPC1 models [14, 57, 72, 73], thus upregulation of PARK7 in individuals with NPC1 may reflect a protective cellular response. We observed a modest elevation in the mean CSF PARK7 level and there was significant overlap of individual values with our non-NPC1 samples. This likely limits the utility of PARK7 as a biomarker for NPC1.

CALB2 is an intracellular calcium binding protein which is highly expressed in cortical interneurons and cerebellar granule neurons. It is essential for normal cerebellar function [74]. CALB2 is a member of hexa-EF-hand protein family, along with CALB1 (Calbindin D) and secretagogin (SCGN). All three of these proteins appear to be elevated in CSF from individuals with NPC1. CALB1 is highly expressed in Purkinje neurons and CSF CALB1 levels are markedly elevated in CSF from NPC1 cats and humans [37]. Increased CALB1 CSF levels are likely indicative of cerebellar Purkinje neuron loss, a major neuropathological finding in NPC1. This study confirmed that CALB1 CSF levels are significantly elevated (*p* = 0.0162, FC = 1.67) in individuals with NPC1. This study also identified a significant elevation of SCGN (*p* = 0.0041, FC = 1.96) in NPC1 CSF. SCGN is highly expressed in the molecular layer of the human, but not rodent, cerebellum and in the hippocampus (reviewed in [75]). SCGN appears to function as a calcium sensor participating in the regulation of exocytosis. Increased NPC1 SCGN levels, like CALB1, may reflect cerebellar dysfunction or damage. Thus, SCGN would be a good candidate biomarker for future validation and characterization. In this study, our PEA screen identified CALB2 as being significantly and markedly (p = 0.0006, FC = 3.41) elevated. This was validated by orthogonal ELISA assay (*p* < 0.0001, FC = 4.2). Data from this study suggest that CALB2 may be a clinically relevant biomarker for NPC1. A strong positive correlation was observed between ASIS and CALB2 NPX values and a moderate positive correlation was observed with our ELISA data. We did not observe an effect of miglustat on this correlation; however, definitive conclusions are precluded by the relatively small number of samples evaluated in this study. A moderate negative correlation was observed with age of neurological onset. Although a larger study is necessary to substantiate these findings, correlation with age of neurological onset and ASIS could have prognostic value.

CHI3L1, also referred to as YKL-40, is a secreted glycoprotein that is a biomarker for neuroinflammation in neurodegenerative disorders such as multiple sclerosis [76] and Alzheimer disease (reviewed in [77]. In the central nervous system, CHI3L1 is expressed in activated astrocytes. In Alzheimer disease CSF CHI3L1 levels are elevated prior to development of cognitive symptoms and are predictive of clinical severity. It has been postulated that CHI3L1, which lacks enzymatic activity, functions as a neuroinflammatory signaling molecule involved in mediating microglia/astrocyte interactions. Our data clearly show that CSF CHI3L1 levels are elevated in individuals with NPC1 with the mean level being 3.2-fold increase above mean non-NPC1 control value. CHI3L1 NPX levels showed a strong positive correlation (*p* = 0.0002, rho = 0.83) with the concurrent NPC1 Neurological Severity score. In this data set, no difference between no miglustat and miglustat samples was observed. The positive correlation was also observed in the ELISA validation set; however, the correlation was moderate (*p* = 0.0183, rho = 0.40). CSF CHI3L1 levels did not significantly correlate with either age of neurological onset or ASIS, suggesting that CHI3L1 will not be useful for prognosis. However, correlation with the current NPC NSS suggest that CHI3L1 may be a biomarker for the current disease state.

Migration Inhibitory Factor (MIF) is an early-stage inflammatory mediator and historically was the first identified cytokine. MIF functions in innate immunity and levels are elevated in sepsis, autoimmune diseases and cancer [78]. MIF is expressed by both neurons and glia and is elevated in CSF from individuals with Alzheimer disease [79]. Our data show that MIF protein is increased in NPC1 CSF relative to non-NPC CSF samples. Although NPC1 CSF levels were elevated, based on a relatively small sample set, they did not correlate well with NPC1 clinical phenotype. However, given its function in initiating inflammatory responses, it may be a target for therapeutic intervention.

CCL18, or CC motif chemokine ligand 18, is a human specific chemokine produced by macrophage and microglia. CCL18 is most closely related to CCL3 [80], another chemokine that has been shown to be elevated in NPC1 CSF ([57, 81] and this study). CCL18 has previously been shown to be elevated in serum/plasma from individuals with lysosomal diseases, specifically Gaucher disease [82], Niemann-Pick disease type B or acid sphingomyelinase deficiency [83], GM1 gangliosidosis [84], NPC [40] and possibly lysosomal acid lipase deficiency [85]. CCL18 clearly functions in multiple different pathological conditions, such as tumor associated macrophages [86] and interstitial lung disease [87], in addition to lysosomal diseases. However, it is interesting to note that all of the above lysosomal disease includes abnormal lipid storage, thus raising the possibility of some degree of specificity. Perhaps consistent with this observation, CCL18 is highly expressed in macrophage present in atherosclerotic plaques [88]. Although not specific for NPC1, CCL18 appears to be a clinically relevant biomarker for NPC1. NPX values for CCL18 indicated a 7.4-fold increase over non-NPC1 samples and 7.3-fold increase was observed in the ELISA validation data. Correlation of the NPX data with clinical parameters showed a strong negative correlation (*p* = 0.0101, rho = -0.75) with age of neurological onset. This strong negative correlation (*p* = 0.0016, rho = -0.65) was also observed when evaluating samples from NPC1 individuals with disease onset prior to age 20 years. Similar to CALB2 we also observed a strong positive correlation (*p* = 0.0017, rho = 0.61) between CSF CCL18 levels and ASIS. These data support the idea that CCL18 may be a prognostic indicator of NPC1 disease severity.

### Identification of novel CSF protein biomarkers with decreased levels in NPC

Only eight proteins were identified with decreased expression (Table 4). As noted above this may be a sensitivity issue. Regulation of lipase activity was identified by pathway analysis, and this is likely due primarily to decreased expression of LPL. The potential role of LPL in NPC1 neuropathology is not clear. LPL is generally thought to be neuroprotective, and polymorphisms have been associated with Alzheimer disease [89]. However, as speculated above, decreased levels could limit neuronal uptake of exogenous cholesterol and thus be a protective response. SPINK6 or Serine Peptidase Inhibitor Kazal Type 6 also appears to be decreased (adj. *p* = 0.0285, FC = 0.72) in CSF from individuals with NPC1. Not much appears to be known about the function of this protein in the brain, but it is interesting to note that it is highly, and compared to cortical neurons relatively specifically, expressed in cerebellar Purkinje neurons (https://www.proteinatlas.org/ENSG00000178172-SPINK6/tissue/cerebellum), thus perhaps reflecting the loss of Purkinje neurons, a characteristic pathological finding in NPC1.

### Study limitations

This study has several limitations. First, obtaining true control pediatric CSF samples is precluded by ethical and regulatory considerations. Adult control samples would raise the concern of age effects. Thus, we compromised by using three independent non neurodegenerative disease cohorts. Although consistent with the fact that NPC1 is an ultrarare disorder, this is a relatively small study. The small number of samples likely impacted our power to detect clinical correlations using the NPX data. The PEA screen is limited to the 1536 proteins that are included in the assay. Since these panels are pre-selected to reflect specific biological pathways of interest, this PEA screen is not unbiased. Theoretically, detection of a specific analyte could be limited by variable protein modifications that impair antibody binding. Although we obtained information on 1467 proteins, an unbiased mass spectrometry screen may yield information on a larger set of proteins. A recent study has suggested that ventricular volume may affect CSF protein concentration [30], thus this may need to be considered as a potential confounder in neurodegenerative diseases such as NPC1. The clinical correlations observed with the ELISA data, and the corresponding conclusions as to their potential value, will need to be confirmed in a larger sample set. Although these limitations need to be considered, this study did identify several biomarker candidates that appear to provide clinically relevant information.

### Potential CSF protein biomarkers and future directions

Our validation efforts were not exhaustive and multiple proteins identified in the PEA screen should be investigated further. Validation of 6-pyruvoyl-tetrahydropterine synthase (PTS) should be considered. PTS is one of three enzymes involved in the biosynthesis of tetrahydrobiopterin (BH4). Neither of the other two enzymes, GTP cyclohydrolase 1 (GCH1) nor sepiapterin reductase (SPR) were assessed by the PEA panel. BH4 is an essential cofactor involved in synthesis of aromatic amino acids (phenylalanine and tyrosine), nitric oxide and neurotransmitters such as serotonin and dopamine. Expression of neuronal oxide synthase (NOS1) and dopamine decarboxylase (DDC) were also upregulated, perhaps suggesting a disease related alteration in these biochemical pathways. Of potential interest, PTS NPX values showed a strong positive correlation (*p* = 0.0052, rho = 0.79) with ASIS values.

Among the top 50 proteins showing increased expression, ranked by fold-change (Table 3), several proteins identified in the PEA screen of NPC1 CSF have been identified in other neurodegenerative diseases. These includes tauopathies involving MAPT [90] and CHI3L1 as discussed above. NEFL and ENO2 are known markers of neuronal injury. PARK7 or DJ-1 was initially implicated in Parkinson disease but has been used as a biomarker for other neurodegenerative disorders [91]. Elevated levels of PDCD5, ENO1, CHI3L1, PP3R1 and SCRN1 have been observed in CSF from individuals with Alzheimer disease [92]. SCRN1 or secernin-1 is interesting since it binds to phosphorylated tau in neurofibrillary tangles present in Alzheimer but not other non-Alzheimer disease tauopathies [93]. Neurofibrillary tangles have been reported in NPC1 brains [94], and it would be of interest to determine whether SCRN1 is a component of NPC1 neuropathology.

This study is the first to identify and confirm increased levels of PARK7, CALB2, CHI3L1, CCL18, MIF and ENO2 in CSF from individuals with NPC1. This study also identified correlations of clinical outcome measures with these proteins. These data can be used to prioritize future studies evaluating a specific protein analyte in a larger cross-sectional and longitudinal sample set. Analysis of longitudinal samples, where an individual can serve as their own control, will allow for both correlation with clinical progression and response to therapeutic interventions.

## Conclusions

In this project we used a PEA screen to identify candidate protein biomarkers with altered CSF expression in individuals with NPC1. To substantiate the utility of this screen, we then validated the increased expression of PARK7, CALB2/calretinin, CHI3L1/YKL-40, CCL18 and ENO2. Interestingly CSF CHI3L1 correlated with the concurrent NPC NSS, suggesting that it may be an indicator of the current disease state. We further demonstrate that CALB2 and CCL18 correlate with age of neurological onset and multiple aspects of the NPC1 phenotype, suggestive of potential utility as prognostic biomarkers. Future work with a larger cohort of samples will be needed to substantiate these observations. As discussed above, the PEA screen also identified a number of other promising targets for validation and correlation with clinical phenotype. Identification of proteins whose expression is altered in a disease state not only have the potential to provide insight into pathological processes but also have the potential to be used to monitor disease progression and therapeutic responses. As such they have the potential to provide additional data supporting a drug’s therapeutic efficacy.

## Supplementary Information

**Additional file 1:** **Figure 1.** Principal component plot of the comparison non-NPC1 samples. The two pediatric laboratory samples that were excluded as outliers are indicated in red.

**Additional file 2:** **Figure 2.** ELISA results for FABP5. For NPC1 samples open circles correspond to individuals who were on miglustat.

**Additional file 3:** **Figure 3.** ELISA results for CALB2, CHI3L1, MIF, CCL18 and FABP5 separated by miglustat therapy status. P-values are from unpaired, two-sided t-tests.

**Additional file 4:** **Table 2.** O-link data analysis. This Excel table provides the adjusted p-values and NPX fold-change for all 1467 proteins relative to comparison samples for both the comparison using only the non-miglustat treated NPC1 samples (columns B and C) and all NPC1 samples (columns D and E). Columns F, G, H and I indicate whether a protein is increased or decreased. The table also indicates top 50 proteins for both analysis (columns J, K, L)

**Additional file 5:** **Table 3.** Individual NPX values for all analytes and samples.

**Additional file 6:** **Table 1.** Sample identification, miglustat status, age and sex.
**Additional file 7:** **Table 4. **CSF proteins that correlate with age.

## Data Availability

Raw data corresponding to the PEA analysis is provided as supplementary data. Anonymized or coded clinical data is available for IRB approved research related to NPC upon request.

## Abbreviations

ASIS: Annual Severity Increment Score
CSF: Cerebrospinal fluid
CTD: Creatine transport deficiency
ELISA: Enzyme-Linked Immunosorbent Assay
FC: Fold change
FDA: Food and Drug Administration
FDR: False discovery rate
HPβCD: 2-Hydroxypropyl-β-cyclodextrin
NPC: Niemann-Pick disease, type C
NPC1: Niemann-Pick disease, type C1
NPC2: Niemann-Pick disease, type C2
NPX: Normalized protein expression
NSS: Neurological Severity Score
PEA: Proximal Extension Assay
PLC: Pediatric laboratory control
PPCS: N-palmitoyl-O-phosphocholineserine
SLOS: Smith-Lemli-Opitz syndrome
