# Supplementary material for: Identification of cerebral spinal fluid protein biomarkers in Niemann-Pick disease, type C1
Source: Biomark Res. 2023 Jan 31;11:14. doi: 10.1186/s40364-023-00448-x (PMC9887810; doi:10.1186/s40364-023-00448-x)
Supplement: Supplementary file 1 — Additional file 1: Figure 1. Principal component plot of the comparison non-NPC1 samples. The two pediatric laboratory samples that were excluded as outliers are indicated in red. [file 40364_2023_448_MOESM1_ESM.pdf]

Additional Figure 1

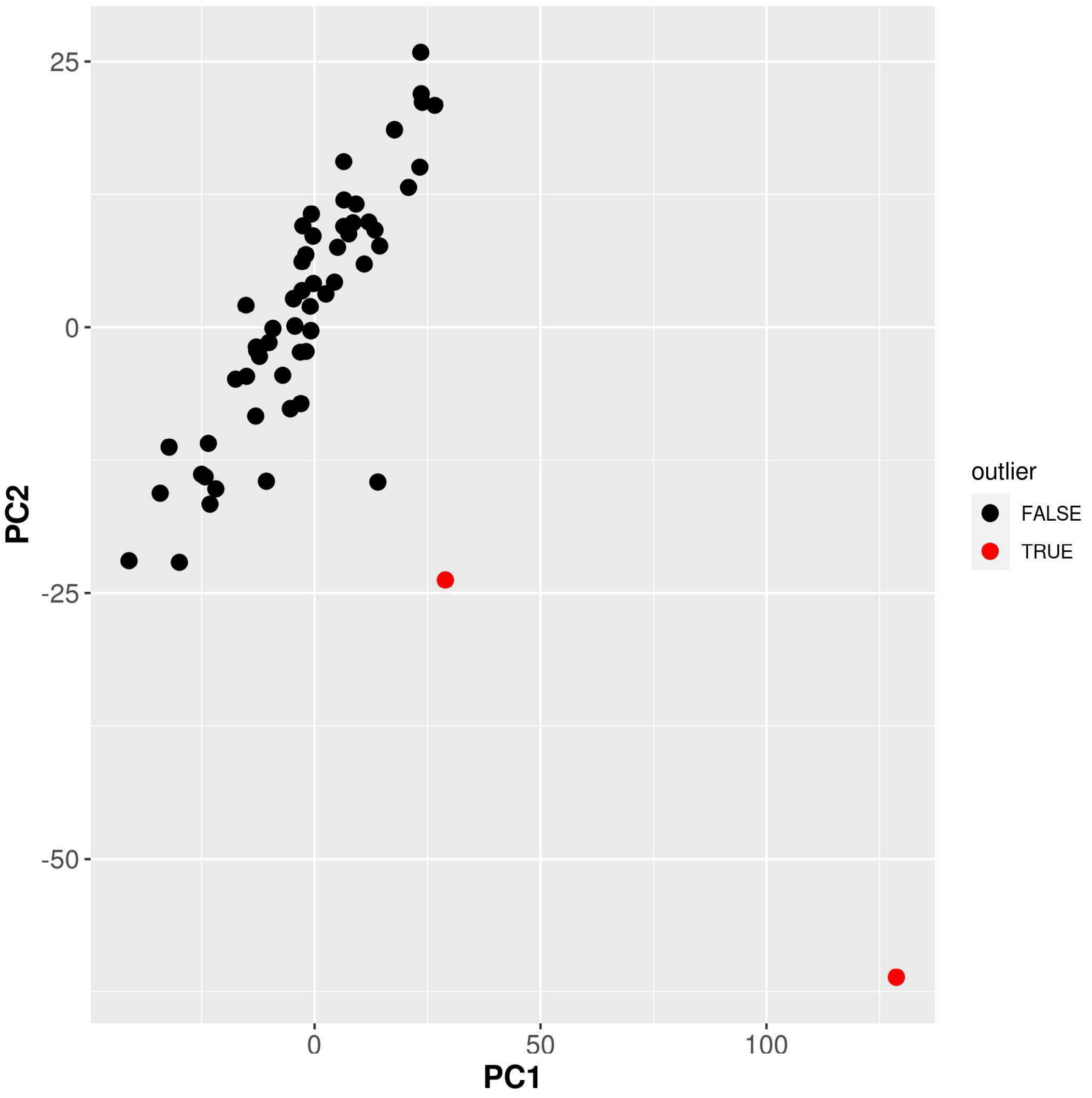

Additional Figure 1. Principal component plot of the comparison non-NPC1 samples. The two pediatric laboratory samples that were excluded as outliers are indicated in red.
