# Supplementary material for: Identification of cerebral spinal fluid protein biomarkers in Niemann-Pick disease, type C1
Source: Biomark Res. 2023 Jan 31;11:14. doi: 10.1186/s40364-023-00448-x (PMC9887810; doi:10.1186/s40364-023-00448-x)
Supplement: Supplementary file 2 — Additional file 2: Figure 2. ELISA results for FABP5. For NPC1 samples open circles correspond to individuals who were on miglustat. [file 40364_2023_448_MOESM2_ESM.pdf]

Additional Figure 2

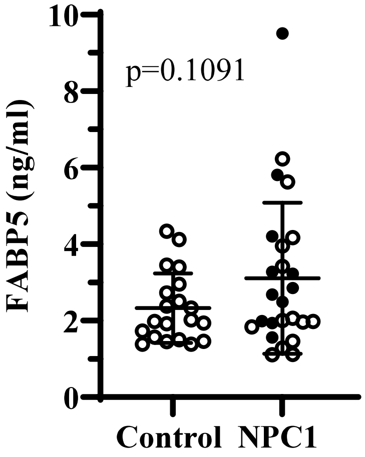

Additional Figure 1. ELISA results for FABP5. For NPC1 samples open circles correspond to individuals who were on miglustat.
