# Supplementary material for: Identification of cerebral spinal fluid protein biomarkers in Niemann-Pick disease, type C1
Source: Biomark Res. 2023 Jan 31;11:14. doi: 10.1186/s40364-023-00448-x (PMC9887810; doi:10.1186/s40364-023-00448-x)
Supplement: Supplementary file 3 — Additional file 3: Figure 3. ELISA results for CALB2, CHI3L1, MIF, CCL18 and FABP5 separated by miglustat therapy status. P-values are from unpaired, two-sided t-tests. [file 40364_2023_448_MOESM3_ESM.pdf]

Additional Figure 3

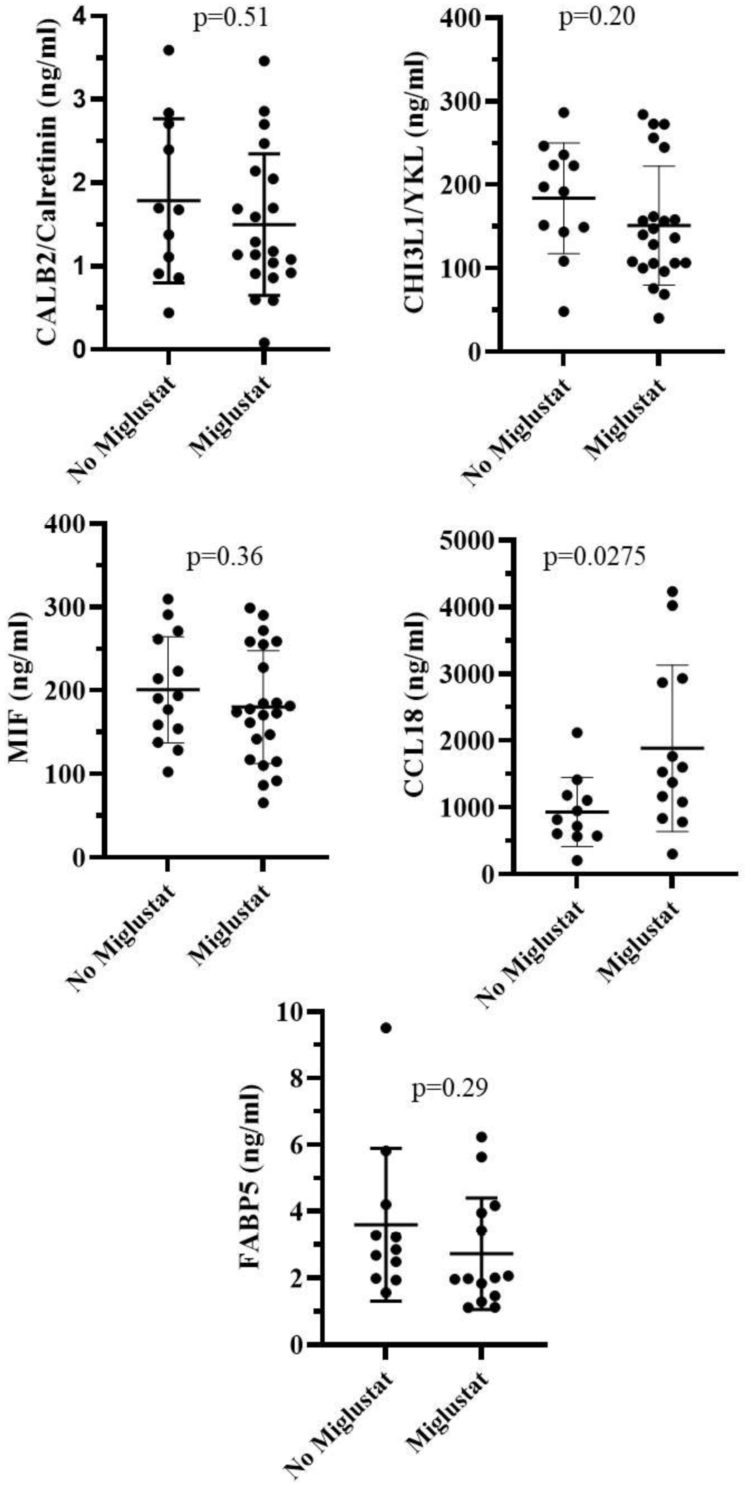

Additional Figure 2. ELISA results for CALB2, CHI3L1, MIF, CCL18 and FABP5 separated by miglustat therapy status. P-values are from unpaired, two-sided t-tests.
